# Dental Treatment Needs and Management in Medically Compromised Children at a Tertiary Pediatric Dental Center—A Retrospective Observational Study

**DOI:** 10.3390/jcm15207763

**Published:** 2026-10-09

**Authors:** Paula Piekoszewska-Ziętek, Dorota Olczak-Kowalczyk

**Affiliations:** Department of Paediatric Dentistry, Medical University of Warsaw, St. Binieckiego 6, 02-097 Warsaw, Poland; pedodoncja@wum.edu.pl

**Keywords:** anesthesia, general, dental care, dental care for Chronically Ill, pediatric dentistry

## Abstract

**Background**: Medically compromised pediatric patients often present with extensive dental treatment needs and may require treatment under general anesthesia due to behavioral limitations or systemic health conditions. The aim of this study was to evaluate dental treatment needs, clinical status, and indications for general anesthesia among medically compromised children referred for dental consultation from a tertiary children’s hospital. **Methods**: This retrospective study included 215 medically compromised children referred from a tertiary children’s hospital to the Department of Pediatric Dentistry between 2020 and 2024. Data extracted included age, sex, type of dentition, systemic diagnosis, dmft/DMFT and pufa/PUFA (the clinical consequences of untreated dental caries, including pulpal involvement, ulceration, fistula, and abscess formation), treatment type, and reasons for the use of general anesthesia. Group comparisons were performed using the Mann–Whitney U test for continuous variables and the chi-square or Fisher’s exact test for categorical variables. **Results**: A total of 62 patients (28.8%) required treatment under general anaesthesia, whereas 153 patients (71.2%) were treated in the outpatient setting. Children treated under general anaesthesia were significantly younger (6.13 vs. 10.60 years; *p* < 0.001) and generally presented with a greater burden of untreated dental disease, with the most pronounced differences observed in primary and mixed dentition. They underwent more extractions and restorative procedures than outpatients. The most common reasons for general anaesthesia were lack of cooperation and extensive dental treatment needs, often co-occurring with systemic comorbidities. **Conclusions**: Among medically compromised children in this tertiary referral cohort, those treated under general anaesthesia generally presented with a greater burden of untreated dental disease, particularly in the primary and mixed dentition groups. These findings support the importance of timely dental assessment and interdisciplinary care in this population.

## 1. Introduction

Children with systemic conditions—such as congenital heart defects, neurological disorders, nephropathies, or malignancies—often present with increased risk of oral health complications, delayed dental care, and complex treatment needs. Depending on the underlying disease, these patients may require modifications in dental management related to behavioral limitations, immunosuppression, antibiotic prophylaxis, bleeding risk, or the need for interdisciplinary medical supervision. The dental management of these medically compromised patients requires a comprehensive, interdisciplinary approach that considers both the systemic disease and the specific oral condition [1].

According to Demir et al. [2], approximately 5% of pediatric dental patients present with systemic comorbidities, most commonly neurological, genetic, or cardiac disorders. These children are often less cooperative, have higher caries experience, and are at increased risk of infection, which complicates both diagnosis and treatment planning.

In the literature, the necessity of using general anesthesia in children with disabilities or behavioral management challenges is frequently emphasized. A retrospective analysis by Bardakçı et al. [3] revealed that epilepsy was the most common condition among children undergoing such procedures. Several previous studies have examined dental treatment provided under general anesthesia in pediatric patients with systemic comorbidities. Previous studies have investigated factors associated with dental treatment under general anaesthesia, including indications for treatment, patient medical characteristics, and the scope of dental procedures performed [4,5,6]. Loyola-Rodrigues et al. [7] have also demonstrated notable differences in the duration and extent of dental procedures between healthy children and those with developmental or medical disabilities, underscoring the impact of general health status on oral care delivery.

It has been demonstrated that dental treatment under general anesthesia in a tertiary care hospital setting allows safe and effective care for patients with complex medical conditions, particularly when extensive dental rehabilitation is required and behavioral management is not feasible [8]. Although dental treatment under general anaesthesia enables comprehensive care in a single session, it is associated with a measurable risk of short-term postoperative morbidity. In a prospective observational study of 56 children, postoperative dental pain was reported in 82% of patients and dental bleeding in 23%; both symptoms resolved within 3 days after treatment [9]. Another prospective study including 133 children reported at least one postoperative complaint in 69.9% of patients after 24 h and in 35.3% after 72 h, with coughing, pain, difficulty eating, behavioural changes, and sore throat being the most frequent symptoms [10]. Therefore, treatment under general anaesthesia should be reserved for cases in which conventional outpatient care is not feasible or when the extent of dental treatment justifies comprehensive care under controlled conditions. Further emphasis has been placed on the need to apply individualized treatment strategies in medically compromised pediatric populations. Selecting between pharmacological and non-pharmacological behavior management techniques requires consideration of not only the child’s age and level of cooperation but also their systemic health condition and medical history [11].

Professional guidelines, such as those published by the American Academy of Pediatric Dentistry, emphasize the need for coordinated care and early preventive interventions in children with special health care needs. These children face significant barriers to access and are more likely to experience unmet dental needs compared to their healthy peers [1].

Despite the growing body of literature on dental treatment under general anaesthesia, most studies have focused on children with severe early childhood caries, developmental disabilities, or behavioral management difficulties. Limited data are available regarding medically compromised children referred from tertiary paediatric hospitals, who represent a heterogeneous population with diverse systemic conditions and potentially distinct dental treatment needs. The novelty of the present study lies in the comprehensive characterization of a heterogeneous population of medically compromised children referred from a tertiary paediatric hospital, incorporating systemic diagnoses, dental disease burden, documented indications for treatment under general anaesthesia, and procedures performed. The aim of this study was to compare clinical characteristics, dental treatment needs, and management approaches between medically compromised children managed in the outpatient setting and those treated under general anaesthesia. We hypothesized that medically compromised children treated under general anaesthesia would present with more severe dental disease, greater treatment needs, and a higher burden of untreated caries than medically compromised children managed in the outpatient setting.

## 2. Materials and Methods

The study was submitted to the Bioethics Committee of the Medical University of Warsaw, which acknowledged the project and confirmed that formal ethical approval was not required due to the retrospective nature of the analysis (Document No. AKBE/232/2024; date: 09/09/2024). The requirement for informed consent was waived by the Bioethics Committee, in accordance with institutional regulations and applicable ethical guidelines.

Medical records used in this study were obtained from the Department of Pediatric Dentistry at the Medical University of Warsaw and covered the period from 2020 to 2024. All records of pediatric patients referred from departments of the affiliated tertiary children’s hospital during the study period were considered for inclusion. Patients were included consecutively if they met the predefined eligibility criteria and if the available medical and dental documentation contained all data required for the planned analyses. Records with incomplete information preventing assessment of the study variables were excluded. Because no prospective screening log was maintained, the exact number of records initially reviewed could not be reliably reconstructed retrospectively.

The study was based exclusively on routinely collected clinical documentation; no dental examinations were performed specifically for research purposes. Patients had been examined by dentists working in the Department of Pediatric Dentistry as part of routine clinical care, using the same departmental diagnostic approach throughout the study period. Formal examiner calibration was not performed as a study-specific procedure because the present investigation was a retrospective analysis of previously recorded clinical data rather than a prospective examination-based study.

Baseline dental status and treatment needs were assessed using data recorded at the first dental visit following referral from the children’s hospital. In patients with multiple visits, this first post-referral examination was used for analysis. Consequently, dmft/DMFT and pufa/PUFA indices reflected the patient’s dental status before the analyzed course of dental treatment. Records lacking sufficient information from this baseline examination were excluded. Treatment needs were defined as the individual dental care requirements identified on the basis of the clinical examination documented at the first visit. These were analysed separately from dental procedures subsequently performed, which represented treatment actually delivered during clinical management.

The following variables were extracted from the records: age, sex, referring hospital department, systemic diagnosis, type of dentition, dmft/DMFT and pufa/PUFA indices [12], documented treatment needs, treatment modality (outpatient care or treatment under general anaesthesia), reasons for the use of general anaesthesia, and dental procedures performed. Reasons for the use of general anaesthesia were extracted separately from the clinical records and could include lack of cooperation, extensive treatment requirements, urgency related to planned medical procedures, and relevant medical or behavioural considerations. More than one reason could be recorded for an individual patient. Thus, baseline dental status and treatment needs, factors documented as indications for treatment under general anaesthesia, and procedures actually performed were treated as distinct categories of information. Lack of cooperation was recorded when it was explicitly documented in the clinical records as an indication for treatment under general anaesthesia. Because of the retrospective design of the study, the degree of cooperation could not be quantified more precisely.

Antibiotic prophylaxis was recorded as a binary variable indicating whether a prophylactic antibiotic had been administered before a planned procedure on the basis of a medical indication. Therapeutic antibiotics prescribed for an active infection were not classified as prophylaxis. The specific medical indication for prophylaxis was not recorded as a separate study variable. Statistical analysis was performed using non-parametric methods appropriate for the data distribution and variable types. Differences between categorical variables (e.g., treatment modality, sex, diagnoses, types of dental procedures) were assessed using the chi-square test (χ^2^) or, where appropriate, Fisher’s exact test. For continuous variables (e.g., age, caries indices such as dmft/DMFT and pufa/PUFA), comparisons between two independent groups were conducted using the Mann–Whitney U test. The strength of associations was measured using effect size coefficients such as Cramér’s V for categorical variables and r for non-parametric tests. A *p*-value of <0.05 was considered statistically significant. Means and standard deviations were used for descriptive presentation of continuous variables, whereas non-parametric tests were applied for between-group comparisons where appropriate. The analyses were exploratory and were intended to characterize differences across clinically distinct dental indices and dentition stages rather than to test a single confirmatory hypothesis or identify independent predictors of treatment modality or dental disease severity. Therefore, no formal adjustment for multiple comparisons was applied, and individual *p* values were interpreted together with the corresponding effect sizes and the overall pattern of findings.

## 3. Results

**Figure 1:**
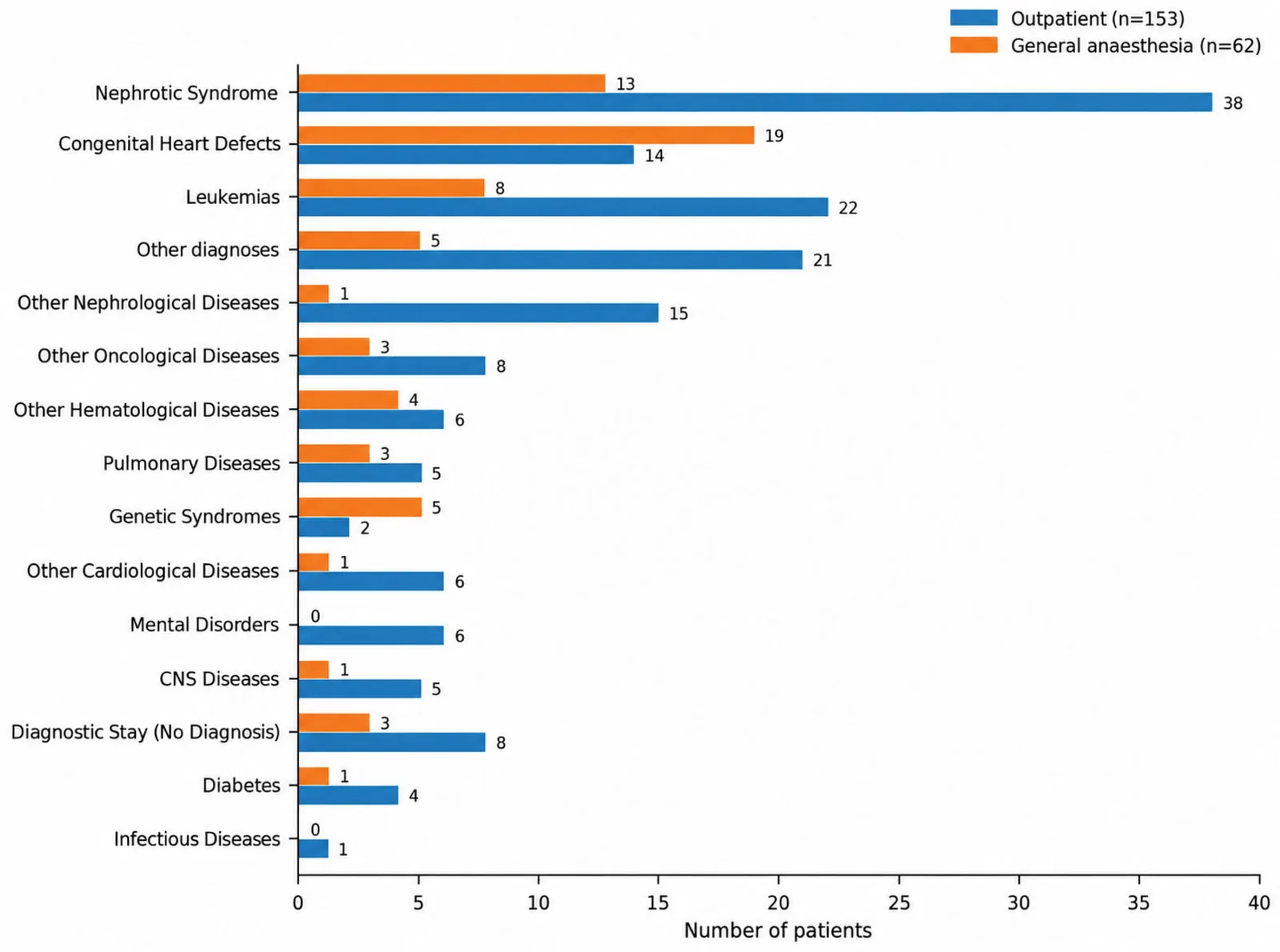
Distribution of systemic diagnoses among referred patients. Patients could have more than one systemic diagnosis; therefore, diagnostic categories were not mutually exclusive.

**Figure 2:**
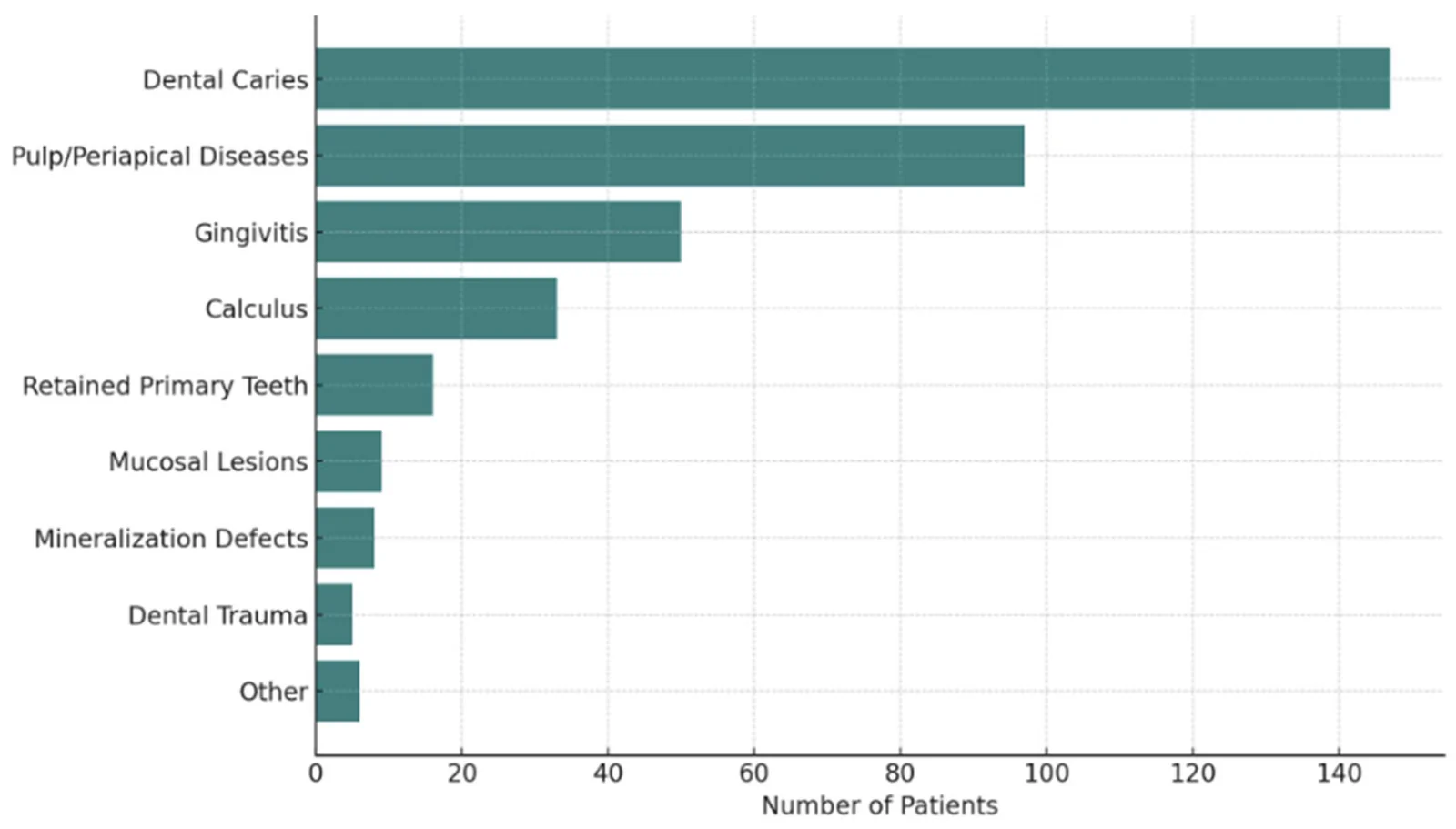
Distribution of dental diagnoses among the study population.

The study group consisted of 215 pediatric patients, including 131 boys (60.9%) and 84 girls (39.1%). The mean age of the entire cohort was 9.31 years (SD = 4.92). No significant differences in sex distribution were observed between the treatment groups, indicating demographic comparability across the groups. The distribution of pediatric patients referred for dental consultation and treatment by each clinical department is presented in Table 1. The study population comprised patients hospitalized for a range of underlying systemic conditions, the distribution of which is presented in Figure 1. The most common dental diagnosis among the study population was dental caries, observed in 68.4% of patients. Other frequently recorded conditions included diseases of the pulp and periapical tissues (45.1%), gingivitis (23.3%), and calculus deposits (15.3%). Less common findings involved retained primary teeth (7.4%), mucosal lesions (4.2%), mineralization defects (3.7%), dental trauma (2.3%), and other miscellaneous diagnoses (2.8%) (Figure 2). In certain cases, multiple diagnoses were documented for individual patients, both for general and dental diseases.

A total of 153 patients (71.2%) were treated in an outpatient setting, while 62 patients (28.8%) received treatment under general anesthesia. In the outpatient group, 94 boys (61.4%) and 59 girls (38.6%) were treated, whereas the general anesthesia group comprised 37 boys (59.7%) and 25 girls (40.3%). No statistically significant differences in sex distribution were observed between the two groups (*p* = 0.811). The mean age of patients treated in the outpatient setting was significantly higher at 10.60 years (SD = 4.76) compared to 6.13 years (SD = 3.74) in the general anesthesia group (*p* < 0.001). This difference in age was consistent across both sexes.

The most frequently reported reason for treatment under general anesthesia was lack of cooperation, followed closely by the presence of numerous teeth requiring treatment or extraction. An urgent need for dental treatment prior to other medical procedures was also a common reason. In several cases, significant systemic comorbidities were documented as one of the indications for treatment under general anaesthesia. Intellectual disability or autism was reported less frequently, and only a single case involved an allergy to local anesthetics. No patients were qualified for general anesthesia solely due to psychiatric disorders or dental phobia. It should be noted that multiple reasons were often present in individual patients. These data are summarized in Table 2.

Analysis of dental status using the dmft/DMFT and pufa/PUFA indices revealed notable differences between patients treated under general anesthesia and those treated on an outpatient basis, with the most pronounced disparities observed in the primary dentition group. The detailed results are presented in Table 3.

Children treated under general anaesthesia consistently exhibited more severe dental disease than those managed in the outpatient setting, particularly in the primary and mixed dentition groups (Table 3). In the primary dentition group, they showed significantly higher caries experience, characterized by a greater number of untreated lesions and more frequent clinical consequences of advanced caries, including pulp involvement and fistula formation. Similar findings were observed in the mixed dentition group, where children treated under general anaesthesia demonstrated a higher burden of untreated disease and significantly greater pufa scores.

Among children with permanent dentition, descriptive differences in oral health indicators were observed between the treatment groups. However, because only three patients with permanent dentition were treated under general anaesthesia, no inferential conclusions were drawn for this subgroup.

The dental treatment procedures performed in the study cohort varied significantly between patients treated in the outpatient setting and those who required general anesthesia. Restorative treatment was provided to 64.7% of patients, while extractions were performed in 51.2% of patients. Individual patients could receive more than one type of dental procedure. These were significantly more frequent in the general anesthesia group, reflecting the need for extensive rehabilitative care in younger or less cooperative patients. Tooth extractions were also notably higher among patients treated under general anesthesia, suggesting a more advanced stage of dental disease in this group. Conversely, endodontic treatments were more prevalent in the outpatient group, possibly due to better cooperation and a higher likelihood of preserving teeth through conservative approaches (Table 4).

Antibiotic prophylaxis was administered in 40.9% of the total study population. The use of antibiotic coverage was slightly more frequent among patients treated under general anesthesia (45.2%) compared to those treated in the outpatient setting (39.2%); however, this difference was not statistically significant (*p* = 0.422).

## 4. Discussion

The presented study examined the dental treatment needs and clinical status of pediatric patients with systemic conditions referred for care to a university-based pediatric dentistry department. By comparing children treated in an outpatient setting with those requiring general anesthesia, the study assessed differences in caries experience, oral health severity, and treatment indicators across primary, mixed, and permanent dentitions. The findings provide insight into the extent of untreated dental disease in this medically compromised population and highlight clinical and behavioural characteristics observed among children treated under general anaesthesia.

Alfarraj et al. [8] performed a retrospective cross-sectional investigation of 730 children with complex medical conditions who underwent comprehensive dental treatment under general anesthesia in a tertiary-care hospital. The authors report that among patients receiving this kind of treatment, the most frequent underlying medical conditions were neurodevelopmental disorders (31%), pediatric oncology (17%), and behavioral disorders such as autism or ADHD (15%). The authors concluded that treatment under general anesthesia constitutes an efficient approach to provide dental care to medically compromised patients, enabling control of general health status and performance of complex dental rehabilitation in a single session. The findings by Alfarraj et al. [8] support our results and emphasize that children with systemic diseases or special health care needs represent a high-risk and high-need group requiring advanced dental management. Moreover, the need for repeat interventions in some cases highlights the chronic nature of oral diseases in this population, accenting the urgency for early preventive dental care strategies.

The systemic or neurologic conditions (e.g., epilepsy) are frequently cited in the literature as a major reason for general anesthesia in children [3,8,13]. A retrospective study by Bardakçı et al. [3] confirmed this observation by analyzing 1666 pediatric patients aged 1 to 15 who underwent dental procedures under general anesthesia. The authors reported that epilepsy was the most common medical condition among these patients, accounting for 13.1% of the cohort. Other frequent diagnoses included cerebral palsy (9.5%), asthma (7.6%), and congenital heart disease (6.6%).

In this study, the most common behavioral indication for dental treatment under general anesthesia was lack of cooperation, a finding consistent with existing literature. Numerous studies have identified uncooperative behavior, whether due to age, anxiety, or developmental limitations, as a primary reason for providing dental care under general anesthesia [8,14]. In contrast, psychiatric conditions or dentophobia alone were not observed as an independent reason in our cohort, confirming results from Bardakçı et al. [3], where such cases were rare or absent. From a medical standpoint, systemic comorbidities—such as nephrological, cardiological, and oncological conditions—were among the most frequent general health indications in our population. This reflects the broad clinical spectrum seen in other studies. For example, Alfarraj et al. [8] reported pediatric oncology and complex medical diagnoses as common underlying factors, while Ivancáková et al. [13] similarly highlighted the role of chronic illnesses and intellectual disabilities in determining the need for general anesthesia. Antibiotic prophylaxis was documented in 40.9% of the study population. This relatively high frequency should be interpreted in the context of a tertiary referral population with substantial medical complexity. Current recommendations restrict antibiotic prophylaxis to selected patients at increased risk of serious infectious complications undergoing relevant invasive dental procedures and emphasize judicious use because of limited evidence, potential adverse effects, and antimicrobial resistance. However, because the specific medical and procedural indications for prophylaxis were not systematically recorded as separate study variables, the present study cannot determine whether individual prescriptions were concordant with current guideline criteria. The observed frequency should therefore be interpreted descriptively rather than as evidence of guideline adherence or overuse. Collectively, these findings indicate that behavioral and systemic health factors were frequently documented as co-occurring indications among children treated under general anesthesia. Also, they underscore the importance of individualized treatment planning and collaboration between dental teams and medical specialists to ensure safe and effective care.

Children treated under general anesthesia generally exhibited a greater burden of untreated dental disease than those managed in the outpatient setting, particularly in the primary and mixed dentition groups. A large study by Andreeva et al. [15] on the status assessment of children treated under general anesthesia reported high dmft/DMFT scores and a predominance of untreated, complicated carious lesions in children managed under general anesthesia, especially in younger age groups. Similarly, Schulz-Weidner et al. [14] reported a high caries experience and substantial treatment burden among preschool and school-aged children with special healthcare needs undergoing dental treatment under general anesthesia.

An important finding of the present study was the lower number of filled teeth and the higher frequency of extractions among children treated under general anesthesia. Several factors may potentially contribute to the higher extraction rates observed among children treated under general anesthesia. Possible explanations include delayed presentation to dental services, competing medical priorities, difficulties in cooperation, and a greater burden of untreated disease at the time of referral. In some medically compromised children, additional considerations such as immunosuppression, concerns regarding infectious complications, or the anticipated prognosis of severely affected teeth may also influence treatment planning. When comprehensive rehabilitation is performed under general anesthesia, clinicians may additionally favor definitive treatment approaches that reduce the likelihood of retreatment and repeated exposure to general anesthesia. However, these factors were not directly evaluated in the present study and should therefore be regarded as possible explanations rather than conclusions derived from our data. From a healthcare system perspective, these findings highlight the importance of integrating preventive dental care into the routine management of medically compromised children. Earlier referral to dental services and closer collaboration between medical and dental providers may facilitate timely detection and treatment of carious lesions, reducing the need for extensive rehabilitation and treatment under general anesthesia. Such strategies could potentially decrease treatment burden, healthcare costs, and the likelihood of repeated dental interventions.

In contrast to some earlier reports that primarily focused on early childhood caries or primary dentition, our inclusion of mixed and permanent dentition underscores the chronic nature of oral disease in at-risk pediatric patients. Given that a previous follow-up study by Ehlers et al. [16] revealed that many children developed new caries and some even required repeat treatment, our findings reinforce the call for long-term preventive strategies and regular follow-up in medically compromised pediatric populations.

An additional concern associated with dental treatment under general anesthesia is the risk of requiring subsequent interventions. Although comprehensive dental rehabilitation performed under general anesthesia may effectively address existing treatment needs, it does not eliminate the underlying factors contributing to disease development. Previous studies have shown that children with severe caries experience remain at increased risk of future dental treatment needs despite extensive rehabilitation. Karl et al. [17] evaluated 1155 pediatric patients treated under general anesthesia and reported that 9% required at least one additional dental rehabilitation procedure under general anesthesia. Children requiring repeated interventions were significantly younger at the time of their first treatment and more frequently had a history of preterm birth or continued use of a baby bottle. The authors emphasized that identifying children at increased risk of repeated general anesthesia is essential for improving long-term treatment outcomes and reducing the burden associated with repeated anesthetic exposure [17]. Similar observations were reported by El Batawi et al. [18], who demonstrated that inadequate control of caries progression and insufficient effectiveness of preventive strategies may increase the likelihood of future treatment under general anesthesia. Their findings emphasize that successful management of high-risk pediatric patients should extend beyond completion of dental procedures and include long-term preventive care, regular follow-up, and active involvement of caregivers [18]. Furthermore, Ehlers et al. [12] found that many children developed new carious lesions following comprehensive dental treatment under general anesthesia despite the initial rehabilitation of existing disease. Collectively, these findings suggest that treatment under general anesthesia should be regarded as one component of an ongoing preventive and therapeutic strategy rather than a definitive solution to oral disease in medically compromised children. Regular dental surveillance, caregiver education, and close interdisciplinary cooperation are essential to reduce recurrent disease and minimize the need for repeated exposure to general anesthesia.

The findings of this study emphasize the substantial weight of untreated dental disease among medically compromised and behaviorally uncooperative pediatric patients, particularly those requiring treatment under general anesthesia. Children treated under general anesthesia generally presented with a greater burden of untreated dental disease and required more invasive procedures, particularly in the primary and mixed dentition groups. These results highlight the importance of early risk identification, timely referral, and interdisciplinary coordination between pediatric medical specialists and dental teams. Given that many of the patients presented with systemic comorbidities and required antibiotic prophylaxis, dental care planning must take into account both behavioral and medical complexities. The data also support the need to expand access to specialized pediatric dental services and to strengthen preventive care protocols, particularly for high-risk children referred from tertiary hospital departments. Although the present study was not designed to establish disease-specific management protocols, the findings suggest that medically compromised children may benefit from earlier dental assessment integrated into routine specialist medical care. Future studies should investigate whether tailored preventive pathways for specific patient groups, such as children with congenital heart disease, chronic kidney disease, or oncological disorders, could reduce treatment burden and the need for general anesthesia.

The present study addresses an important gap in the literature, as most previous investigations have focused on children treated under general anesthesia because of severe early childhood caries, developmental disabilities, or behavioral management difficulties. In contrast, our cohort consisted exclusively of medically compromised pediatric patients referred from a tertiary children’s hospital and represented a broad spectrum of systemic conditions. This design allowed us to characterize both the dental treatment needs and treatment approaches of a clinically heterogeneous population that is rarely evaluated as a whole. The findings showed that children treated under general anesthesia had a greater burden of untreated dental disease in addition to frequently presenting with behavioral management difficulties. Given the retrospective design and potential confounding by age, dentition stage, cooperation level, and systemic disease characteristics, these differences should be interpreted as descriptive associations rather than independent predictors of treatment modality.

This study has several limitations that should be considered when interpreting the results. Its retrospective design relied on the accuracy and completeness of existing medical and dental records, which may have introduced documentation bias and limited control over potentially relevant variables. The study was conducted in a single tertiary-care pediatric dental department, which may restrict the generalizability of the findings to broader pediatric populations or primary care settings. The study population was medically heterogeneous, and many patients presented with multiple coexisting systemic conditions. Consequently, the influence of specific diagnoses on dental treatment needs and treatment modality could not be reliably assessed. Moreover, the absence of a healthy control group limits the scope of interpretation. The analyses were designed to compare medically compromised children treated under general anesthesia with medically compromised children managed in the outpatient setting rather than to evaluate differences between medically compromised and healthy children. Therefore, the findings should be interpreted as reflecting differences related to treatment modality within this specific population and cannot be used to determine the independent impact of systemic disease on oral health outcomes. Several potential confounding factors, including age, type of dentition, cooperation level, and the nature or severity of systemic disease, may have influenced both treatment modality and dental disease severity. As no multivariable adjustment was performed, the observed associations should not be interpreted as evidence of an independent effect of treatment under general anesthesia. The lack of long-term follow-up prevented assessment of treatment outcomes, disease recurrence, and the need for repeated treatment under general anesthesia. In addition, some subgroup analyses involved relatively small numbers of patients, particularly those with permanent dentition treated under general anesthesia. These findings should therefore be interpreted with caution and confirmed in larger multicenter studies.

## 5. Conclusions

In this tertiary referral population, children treated under general anesthesia generally presented with a greater burden of untreated dental disease, particularly in the primary and mixed dentition, and frequently had documented difficulties with cooperation or extensive treatment requirements. These findings support the need for earlier and more structured referral pathways between paediatric hospital departments and specialized dental services, with dental assessment incorporated into the ongoing care of medically compromised children before extensive treatment becomes necessary. Preventive care should include regular dental surveillance, individualized caries-prevention strategies, and caregiver education. Close communication between medical and dental teams is also important when planning invasive dental procedures, antibiotic prophylaxis, and treatment under general anesthesia. These recommendations should be interpreted in the context of a tertiary referral population and require confirmation in larger multicenter studies.

## Figures and Tables

**Table 1:** Distribution of patients by referring hospital department.

Hospital Department	Number of Patients (Percentage)
Hematology and Oncology	47 (21.9)
Nephrology	77 (35.8)
Gastroenterology	6 (2.8)
Cardiology	44 (20.5)
Pneumonology and Allergology	12 (5.6)
Endocrinology	4 (1.9)
Laryngology	5 (2.3)
Neurology	5 (2.3)
Psychiatry	4 (1.9)
Surgery	1 (0.5)
Pediatrics	6 (2.8)
Infectious Diseases	1 (0.5)
Intensive Care	1 (0.5)
Diabetology	2 (0.9)

**Table 2:** Reasons for general anaesthesia among patients treated under general anaesthesia (N = 62).

Indication for General Anaesthesia *	Number of Patients n (%)
Lack of cooperation	23 (37.1%)
Presence of numerous teeth requiring treatment or extraction	22 (35.5%)
Urgent need for dental treatment prior to other medical procedures	17 (27.4%)
Significant systemic comorbidities	9 (14.5%)
Intellectual disability or autism	4 (6.5%)
Allergy to local anesthetics	1 (1.6%)
Psychiatric disorders or dental phobia	0 (0%)

* More than one reason could be recorded for an individual patient; therefore, percentages do not sum to 100%.

**Table 3:** Dental status assessed using the dmft/DMFT and pufa/PUFA indices.

		Overall N = 215	Outpatient 153 N = 153	General Anaesthesia N = 62	Statistical Significance
Primary dentition	N (%)	74 (34.4%)	35 (22.9%)	39 (62.9%)	χ^2^(1) = 31.32; *p *< 0.001 *V* = 0.38
dmft	mean (SD)	6.52 (5.00)	3.44 (3.18)	9.21 (4.76)	*U* = 220; *Z* = −4.93; *p* < 0.001; *r* = 0.58
d (decayed teeth)		5.95 (5.10)	2.35 (2.73)	9.08 (4.60)	*U* = 155; *Z* = −5.66; *p* < 0.001; *r* = 0.66
m (missing teeth)		0.08 (0.36)	0.12 (0.41)	0.05 (0.32)	*U* = 622.5; *Z* = −1.14; *p *= 0.256
f (filled teeth)		0.68 (1.36)	1.06 (1.52)	0.36 (1.14)	*U* = 476; *Z* = −2.69; *p *= 0.007; *r* = 0.31
pufa		1.90 (2.73)	0.62 (1.50)	3.03 (3.07)	*U* = 364; *Z* = −3.60; *p *< 0.001; *r* = 0.42
p (pulp involvement)		1.12 (1.72)	0.38 (0.95)	1.77 (1.98)	*U* = 407; *Z* = −3.31; *p *= 0.001; *r* = 0.39
u (ulceration)		0.01 (0.12)	0 (0)	0.03 (0.16)	*U* = 646; *Z* = −0.1793 *p *= 0.350
f (fistula)		0.81 (1.65)	0.26 (0.9)	1.28 (2.03)	*U* = 485.5; *Z* = −2.50; *p *= 0.012; *r* = 0.29
a (abscess)		0.07 (0.35)	0.03 (0.17)	0.10 (0.45)	*U* = 647.5; *Z* = −0.50; *p *= 0.18
Mixed dentition	N (%)	77 (35.8%)	57 (37.3%)	20 (32.3%)	χ^2^(1) = 0.48; *p *= 0.489
dmft	mean (SD)	5.59 (3.23)	5.14 (2.97)	6.85 (3.65)	*U* = 412.5; *Z* = −1.75; *p *= 0.079
d (decayed teeth)		3.70 (3.44)	3.02 (2.88)	5.60 (4.17)	*U* = 353.5; *Z* = −2.47; *p *= 0.014; *r* = 0.29
m (missing teeth)		0.17 (0.58)	0.21 (0.65)	0.05 (0.22)	*U* = 507; *Z* = −1.12; *p *= 0.265
f (filled teeth)		1.86 (1.84)	2.09 (1.94)	1.20 (1.36)	*U* = 408; *Z* = −1.85; *p *= 0.064
pufa		1.57 (1.93)	1.14 (1.58)	2.75 (2.34)	*U* = 308.5; *Z* = −3.11; *p *= 0.002; *r* = 0.36
p (pulp involvement)		0.75 (1.34)	0.68 (1.30)	0.95 (1.47)	*U* = 516.5; *Z* = −0.62; *p *= 0.533
u (ulceration)		0.01 (0.12)	0.02 (0.13)	0 (0)	*U* = 550; *Z* = −0.60; *p *= 0.550
f (fistula)		0.79 (1.42)	0.46 (1.03)	1.70 (1.92)	*U* = 355; *Z* = −2.95; *p *= 0.003; *r* = 0.35
a (abscess)		0.09 (0.50)	0.11 (0.56)	0.05 (0.22)	*U* = 557.5; *Z* = −0.08; *p *= 0.939
DMFT		2.20 (2.23)	1.84 (2.16)	3.26 (2.13)	*U* = 322; *Z* = −2.72; *p *= 0.006; *r* = 0.32
D (decayed teeth)		1.55 (2.01)	1.28 (1.96)	2.37 (1.98)	*U* = 353; *Z* = −2.42; *p *= 0.015; *r* = 0.28
M (missing teeth)		0.03 (0.16)	0.04 (0.19)	0 (0)	*U* = 522.5; *Z* = −0.82; *p *= 0.411
F (filled teeth)		0.70 (1.20)	0.63 (1.06)	0.89 (1.56)	*U* = 523; *Z* = −0.27; *p *= 0.787
PUFA		0.18 (0.62)	0.18 (0.66)	0.21 (0.50)	*U* = 533; *Z* = −0.20; *p *= 0.839
P (pulp involvement)		0.18 (0.63)	0.18 (0.66)	0.21 (0.54)	*U* = 506; *Z* = −0.80; *p *= 0.424
U (ulceration)		0 (0)	0 (0)	0 (0)	-
F (fistula)		0 (0)	0 (0)	0 (0)	-
A (abscess)		0 (0)	0 (0)	0 (0)	-
Permanent dentition	N (%)	64 (29.4%)	61 (39.5%)	3 (4.8%)	*
DMFT	mean (SD)	5.89 (4.40)	5.72 (4.44)	9.33 (1.16)	-
D (decayed teeth)		3.89 (4.50)	3.68 (4.52)	8 (0)	-
M (missing teeth)		0.10 (0.43)	0.10 (0.44)	0 (0)	-
F (filled teeth)		1.92 (1.86)	1.95 (1.89)	1.33 (1.16)	-
PUFA		0.87 (1.61)	0.73 (1.35)	3.67 (3.79)	-
P (pulp involvement)		0.68 (1.19)	0.62 (1.14)	2 (1.73)	-
U (ulceration)		0.02 (0.13)	0 (0)	0.33 (0.58)	-
F (fistula)		0.05 (0.22)	0.05 (0.22)	0 (0)	-
A (abscess)		0.05 (0.28)	0.05 (0.29)	0 (0)	-

* Inferential statistical comparisons were not performed for the permanent-dentition subgroup because only three patients in this subgroup were treated under general anaesthesia. The data are therefore presented descriptively.

**Table 4:** Dental treatment procedures performed on patients *.

	Overall	Outpatient	General Anaesthesia	Statistical Significance
Dental treatment:				
Restorative	139 (64.7%)	84 (54.9%)	55 (88.7%)	χ^2^(1) = 22.07; *p *< 0.001 *V* = 0.32
Endodontic	18 (8.4%)	17 (11.1%)	1 (1.6%)	χ^2^(1) = 5.19; *p *= 0.023 *V* = 0.16
Extractions	110 (51.2%)	62 (40.5%)	48 (77.4%)	χ^2^(1) = 22.04; *p *< 0.001 *V* = 0.33
Scaling	27 (12.6%)	21 (13.7%)	6 (9.7%)	χ^2^(1) = 0.66; *p *= 0.417
Surgical other than extractions	6 (2.8%)	5 (3.3%)	1 (1.6%)	Exact Fisher Test *p *= 0.675
Specialist consultation	29 (13.5%)	29 (19%)	0 (0%)	χ^2^(1) = 13.58; *p *< 0.001 *V* = 0.25

* Values represent the number and percentage of patients who received at least one procedure of the respective category. Individual patients could receive more than one type of dental procedure; therefore, percentages do not sum to 100%.

## Data Availability

The datasets generated and/or analyzed during the current study are not publicly available due to patient privacy and institutional ethical restrictions. De-identified data may be made available from the corresponding author upon reasonable request and subject to approval by the relevant institutional authorities and ethical regulations.

## References

[1] American Academy of Pediatric Dentistry (2024). Management of dental patients with special health care needs. The Reference Manual of Pediatric Dentistry.

[2] Demir P., Özden Ş.N. (2021). Prevalence of medically compromised children among dental patients: A 10-year retrospective study. J. Pediatr. Res..

[3] Bardakçı E., Yıldız Ş., Yazmacı B., Doğan M.E., Mumcu K., Doğan M.S. (2025). Evaluation of pediatric patients’ general health status prior to dental treatment under general anesthesia: A retrospective study. Children.

[4] Al-Khotani A., Ajabnoor H., Koshak R., Alshehri S.A., Meisha D.E. (2025). Clinical profiles and treatment outcomes of dental rehabilitation in patients treated under general anesthesia: A comparison between healthy and special healthcare needs children. Front. Pediatr..

[5] Groß T., Stocker S.A., Scholz K.J., Has C., Vach K., Cieplik F. (2025). Oral health of patients with epidermolysis bullosa compared to healthy controls—A retrospective study from a specialized center. BMC Oral Health.

[6] Mankovsky B., Halushko O., Zablotskyy Y., Synytsyn M. (2026). General anesthesia in patient with Hutchinson-Gilford Progeria syndrome: Two case reports of dental treatment in the one patient. BMC Anesthesiol..

[7] Loyola-Rodriguez J.P., Zavala-Alonso N.V., Martinez-Castañon G.A., Patiño-Marín N., Martinez-Rider R., Reyes-Macias J.F. (2010). Dental treatment under general anesthesia in healthy and medically compromised/developmentally disabled children: A comparative study. J. Clin. Pediatr. Dent..

[8] Alfarraj J., Gray J.L., Alargan T., Alkathiri M., Alshehri R., Almarwan M. (2024). Dental treatment provided to special needs children under general anesthesia in a tertiary care hospital: A cross-sectional retrospective study. Saudi Dent. J..

[9] Hu Y.H., Tsai A., Ou-Yang L.W., Chuang L.C., Chang P.C. (2018). Postoperative dental morbidity in children following dental treatment under general anesthesia. BMC Oral Health.

[10] Almaz M.E., Oba A.A., Sönmez I.S. (2019). Postoperative morbidity in pediatric patients following dental treatment under general anesthesia. Eur. Oral Res..

[11] Baakdah R.A., Turkistani J.M., Al-Qarni A.M., Al-Abdali A.N., Alharbi H.A., Bafaqih J.A., Alshehri Z.S. (2021). Pediatric dental treatments with pharmacological and non-pharmacological interventions: A cross-sectional study. BMC Oral Health.

[12] Monse B., Heinrich-Weltzien R., Benzian H., Holmgren C., van Palenstein Helderman W. (2010). PUFA—An index of clinical consequences of untreated dental caries. Community Dent. Oral Epidemiol..

[13] Koberova Ivancakova R., Suchanek J., Kovacsova F., Cermakova E., Merglova V. (2019). The analysis of dental treatment under general anaesthesia in medically compromised and healthy children. Int. J. Environ. Res. Public Health.

[14] Schulz-Weidner N., Schlenz M.A., Jung L.G., Uebereck C.F., Nehls A., Krämer N. (2022). Dental treatment under general anesthesia in pre-school children and schoolchildren with special healthcare needs: A comparative retrospective study. J. Clin. Med..

[15] Andreeva R. (2018). Dental status assessment of children treated under general anesthesia. Scr. Sci. Med. Dent..

[16] Ehlers V., Callaway A., Patyna M., Pelkner A., Azrak B., Deschner J. (2022). Oral health of children one to six years after dental treatment under general anaesthesia. J. Clin. Med..

[17] Karl V., Scholz K.J., Hiller K.-A., Tabenski I., Schenke F., Buchalla W., Kirschneck C., Bekes K., Cieplik F. (2022). Retrospective cohort study on potential risk factors for repeated need of dental rehabilitation under general anesthesia in a private pediatric dental practice. Children.

[18] El Batawi H.Y. (2014). Factors affecting clinical outcome following treatment of early childhood caries under general anaesthesia: A two-year follow-up. Eur. Arch. Paediatr. Dent..

